# Supplementary material for: Whole-genome sequencing of 3135 individuals representing the genetic diversity of the Japanese population
Source: J Hum Genet. 2025 Nov 8;71(4):223–30. doi: 10.1038/s10038-025-01430-1 (PMC13021519; doi:10.1038/s10038-025-01430-1)
Supplement: Supplementary file 2 — Supplementary Figures S1,S2,S3,S4,S5,S6 [file 10038_2025_1430_MOESM2_ESM.pdf]

Supplementary figures

Supplementary Figure S1

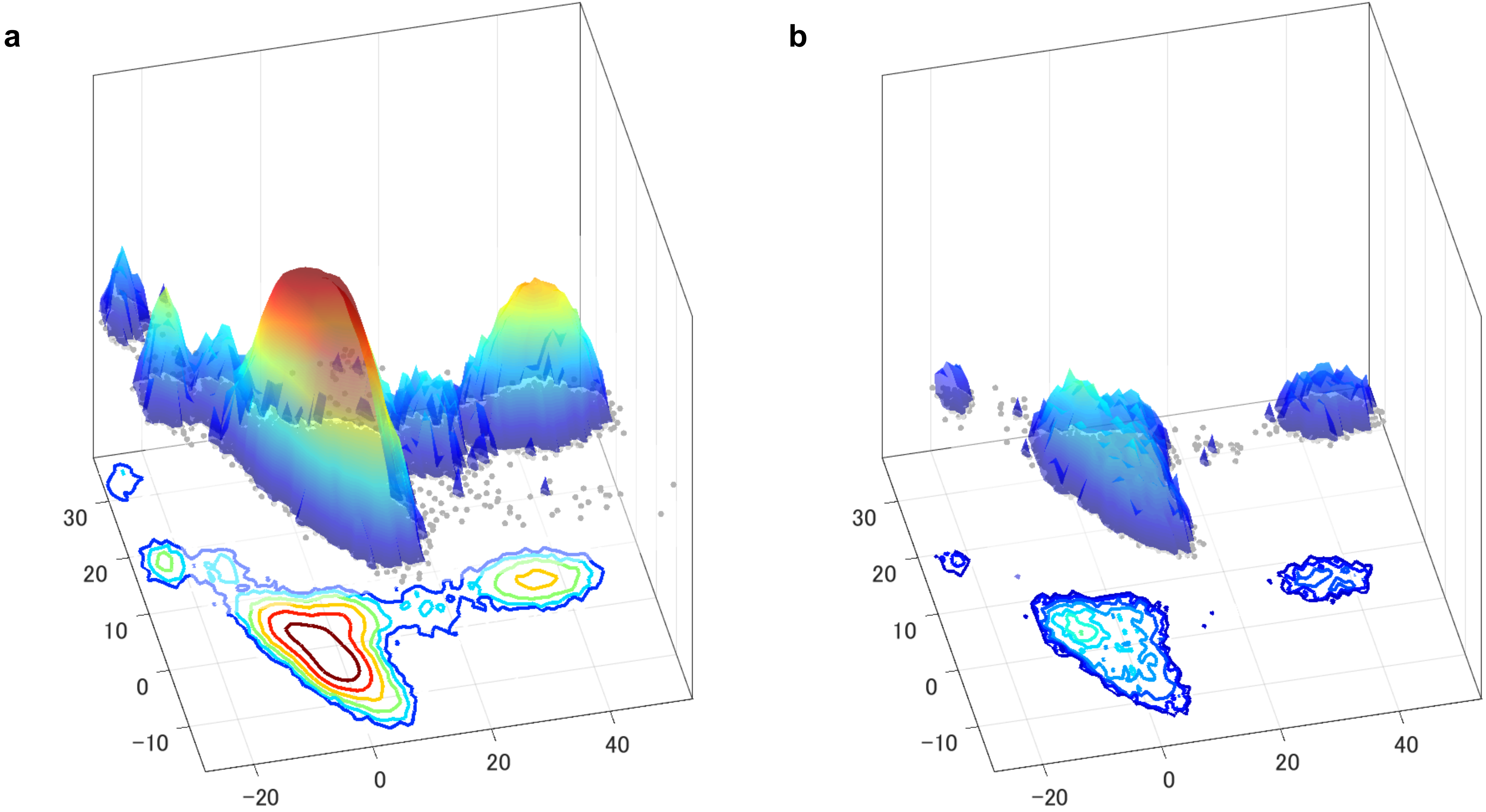

Supplementary Figure S2

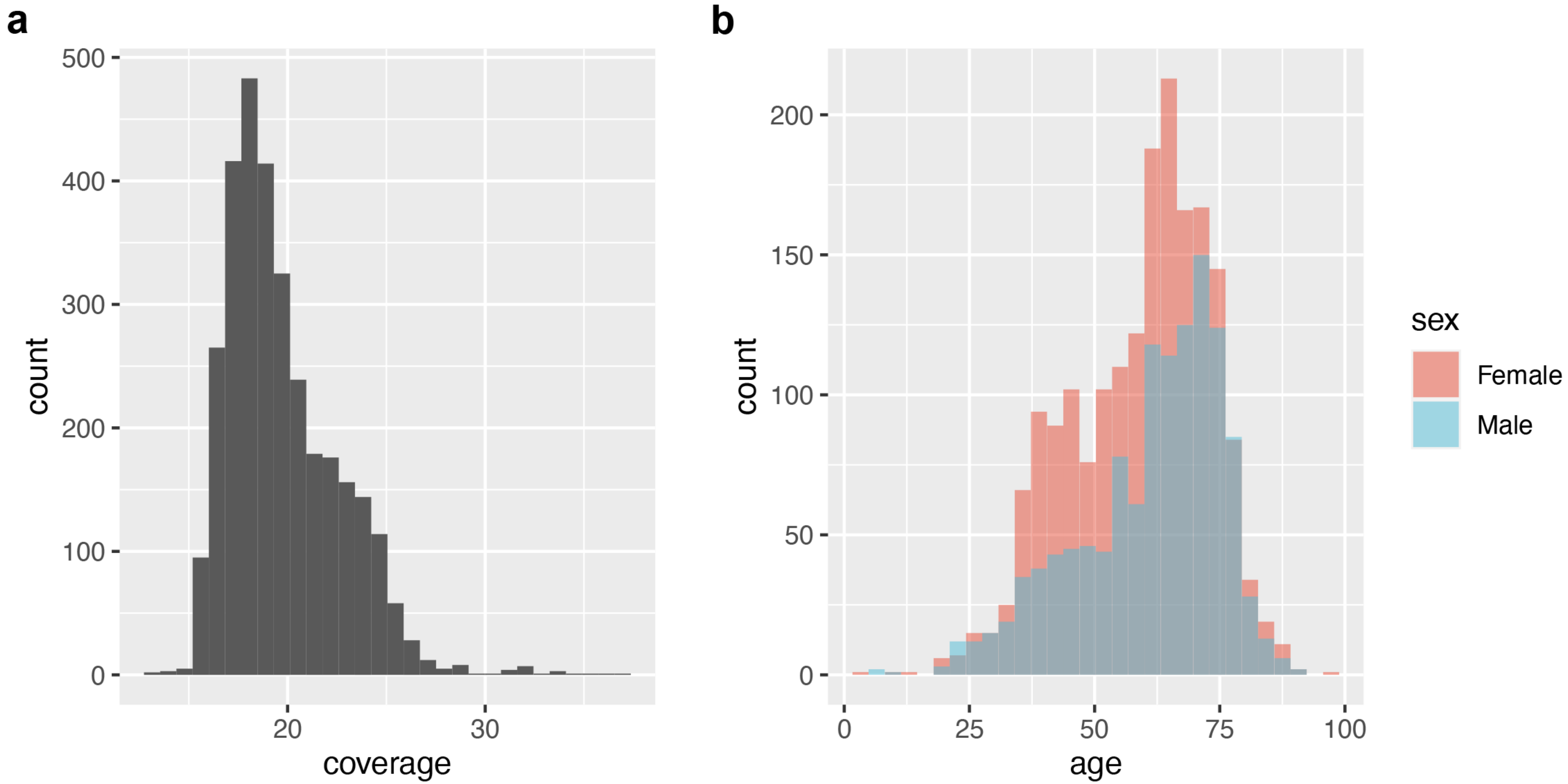

Supplementary Figure S3

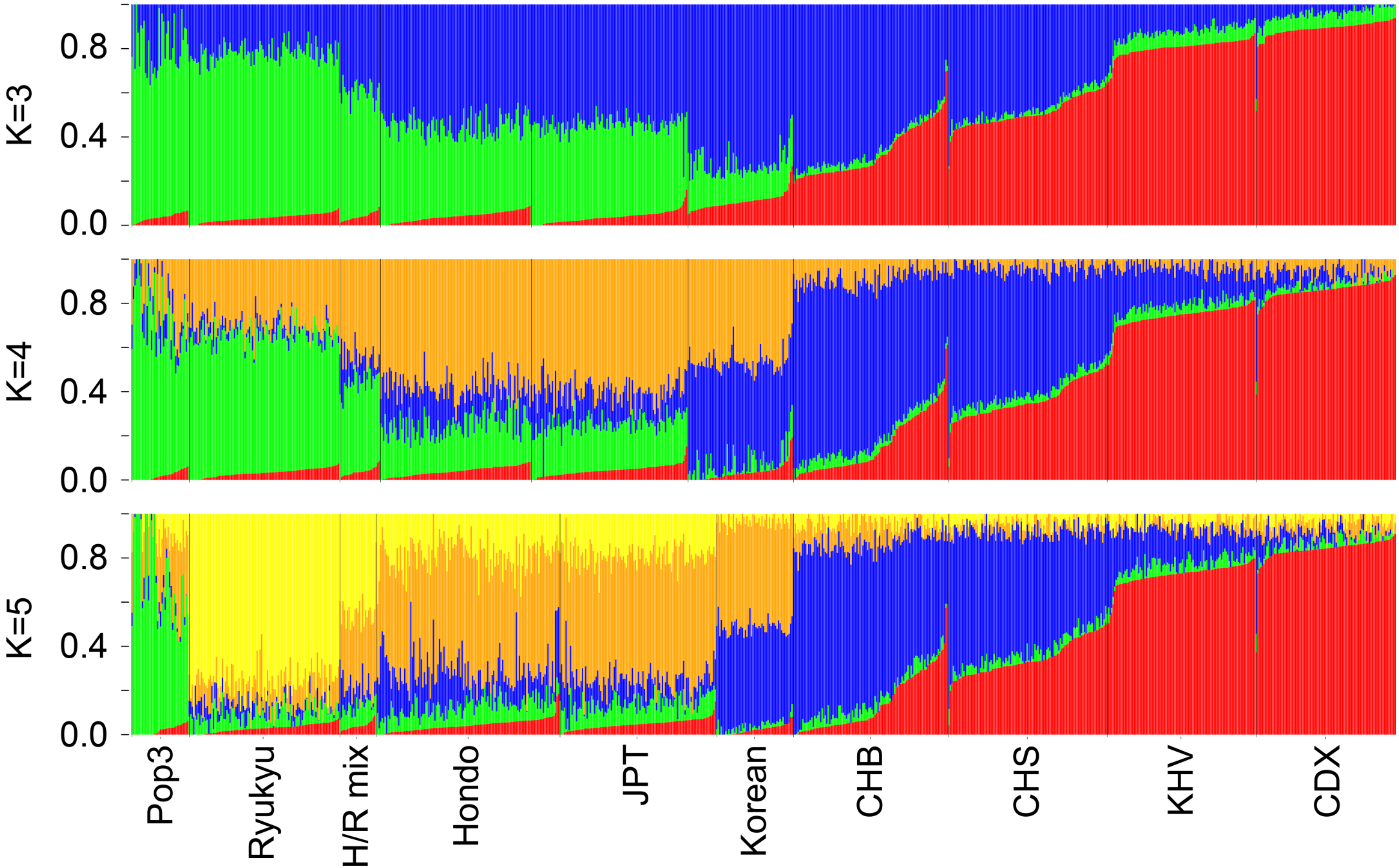

Supplementary Figure S4

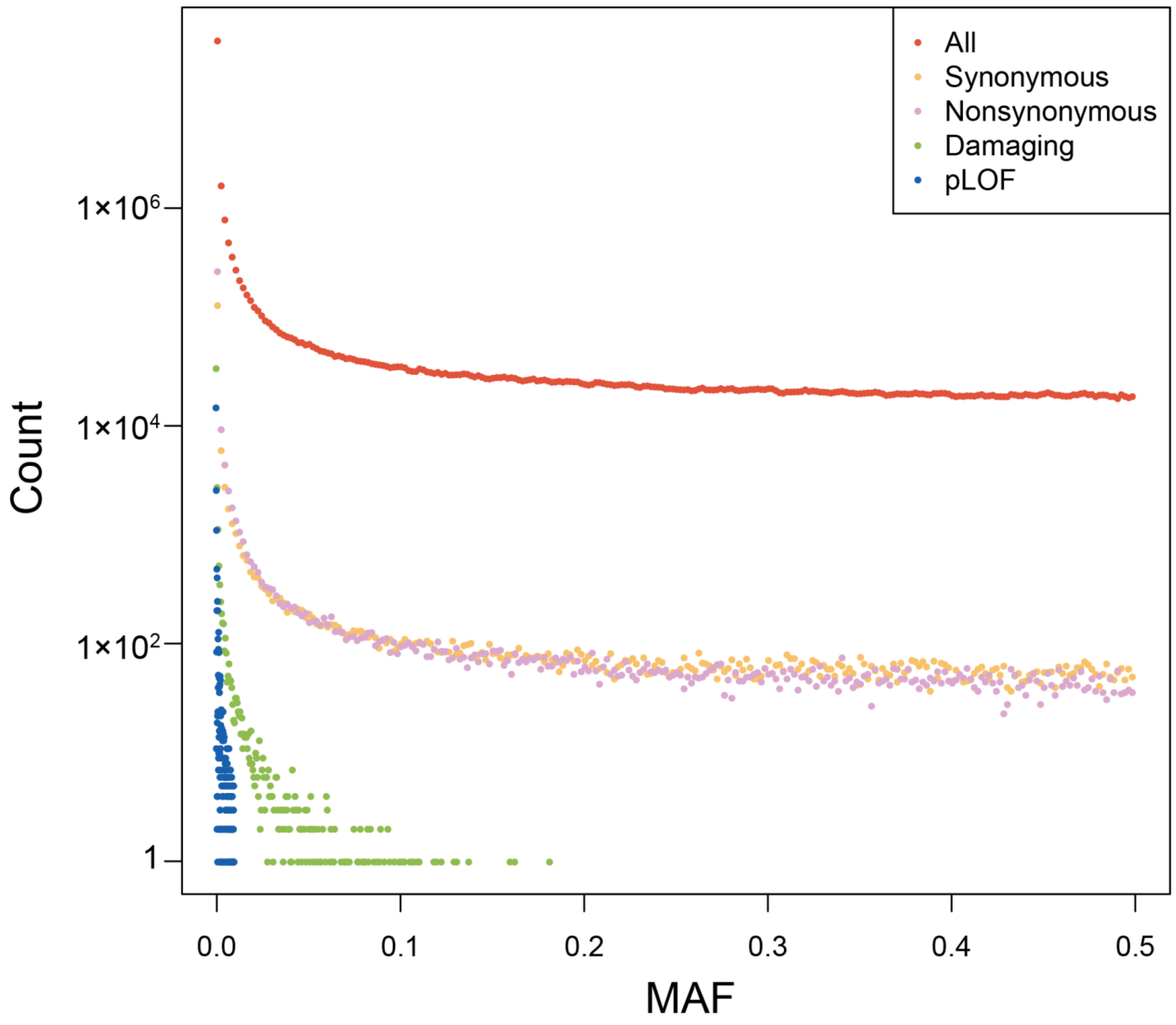

Supplementary Figure S5

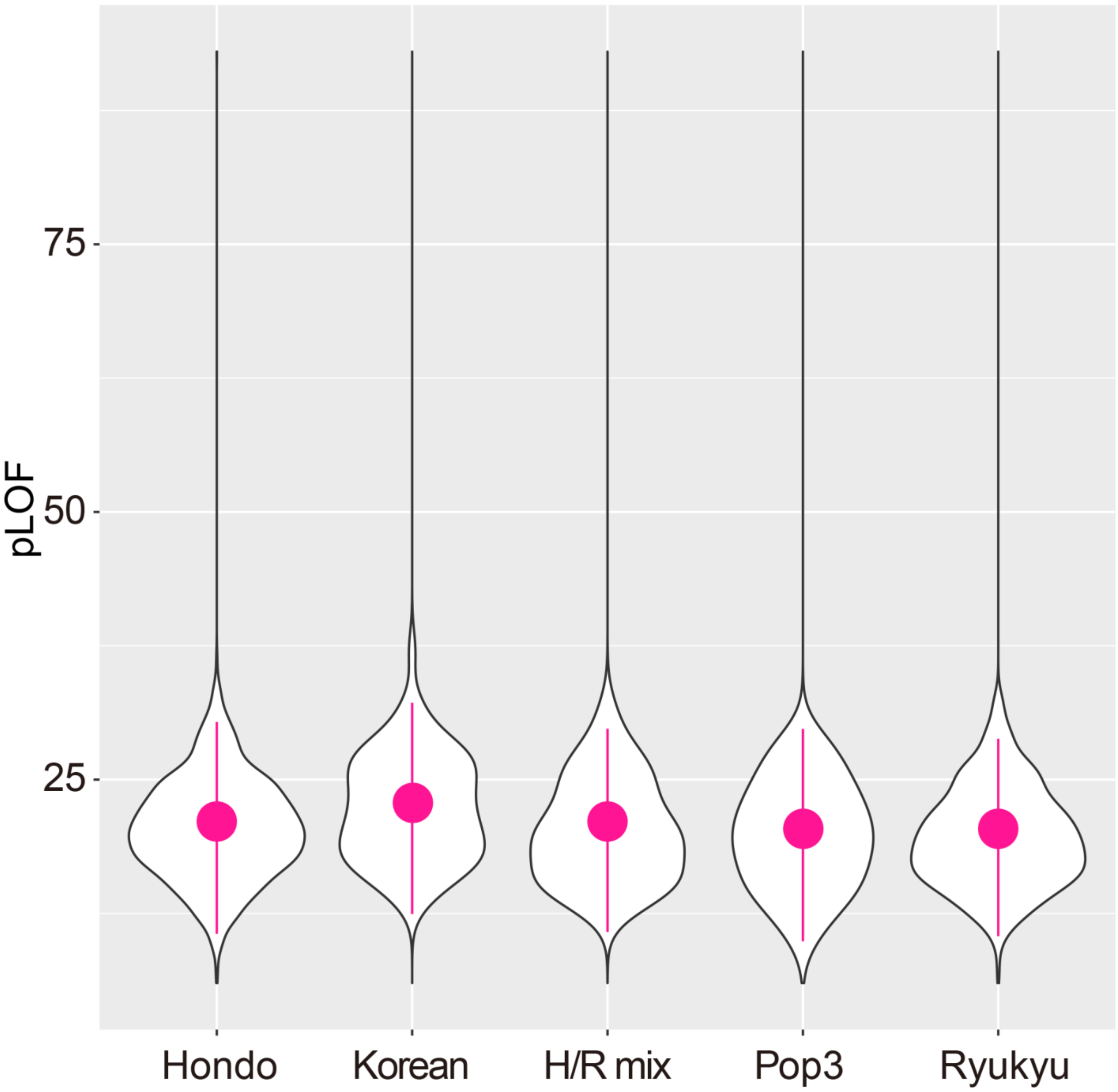

Supplementary Figure S6

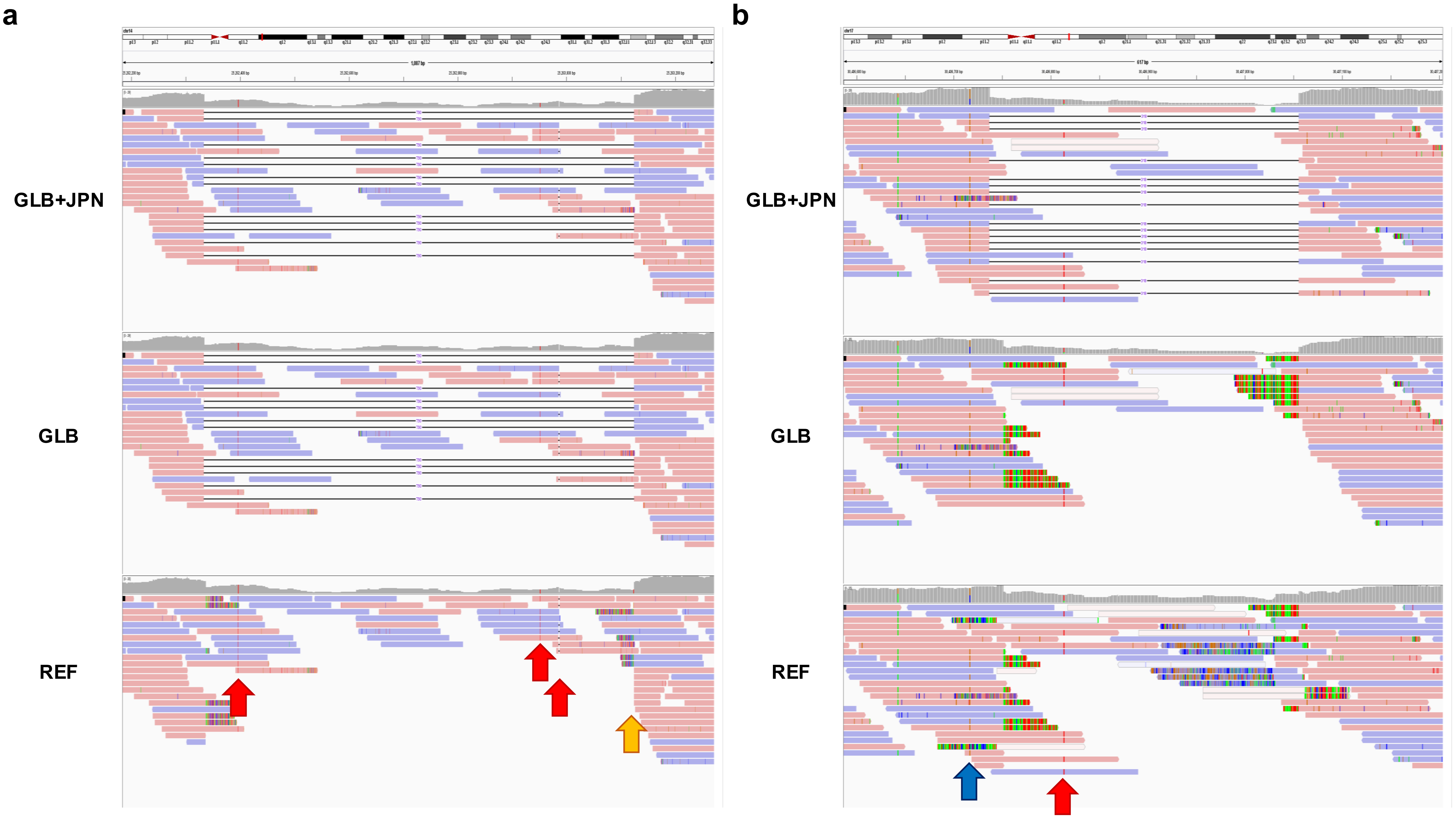

Supplemental information titles and legends

Supplementary Figure S1. Principal component analysis of the Japanese population. The first and second principal components are plotted with densities for (**a**) 178,886 samples and (**b**) 3,148 representative samples of the Japanese population.

Supplementary Figure S2. Basic summary of sequencing across 3,148 individuals. The distribution of (**a**) mean genome-wide coverage and (**b**) age of males and females are shown.

Supplementary Figure S3. ADMIXTURE analysis after adjusting the sample size of each cluster. A total of 100 individuals were selected randomly from each Hondo and Ryukyu cluster to avoid overrepresentation bias. Error rates of five-fold cross-validation for K = 2, 3, 4, and 5 ancestral components were 0.56517, 0.56435, 0.56475, and 0.56566, respectively. JPT: Japanese in Tokyo, Japan, CHB: Han Chinese in Beijing, China, CHS: Southern Han Chinese, KHV: Kinh in Ho Chi Minh City, Vietnam, CDX: Chinese Dai in Xishuangbanna, China.

Supplementary Figure S4. Allele-frequency distributions of variants in each category. The number of variants in synonymous, nonsynonymous, severely damaging and, pLOF categories are shown.

Supplementary Figure S5. Burden of pLOF variants. The number of pLOF variants per individual across the five clusters.

Supplementary Figure S6. Advantages of the reference graph structure. Examples of heterozygous deletion sites at chromosome 14 position 25,261,884–25,263,574 (**a**) and chromosome 17 position 30,436,287–30,437,505 (**b**) are apparent through the analysis with linear (REF), global graph (GLB), and global and Japanese graph (GLB+JPN) references. The GLB+JPN bridges short reads to span SVs in both regions, whereas the GLB only supports region A. Correction of genotypes, increase of supporting reads, and removal of miscalls as observed through analysis of the graph structures, but not the linear reference, are indicated in red, blue, and gold arrows.
